# Potential of Meta-Omics to Provide Modern Microbial Indicators for Monitoring Soil Quality and Securing Food Production

**DOI:** 10.3389/fmicb.2022.889788

**Published:** 2022-06-30

**Authors:** Christophe Djemiel, Samuel Dequiedt, Battle Karimi, Aurélien Cottin, Walid Horrigue, Arthur Bailly, Ali Boutaleb, Sophie Sadet-Bourgeteau, Pierre-Alain Maron, Nicolas Chemidlin Prévost-Bouré, Lionel Ranjard, Sébastien Terrat

**Affiliations:** ^1^Agroécologie, INRAE, Institut Agro, Université Bourgogne, Université Bourgogne Franche-Comté, Dijon, France; ^2^Novasol Experts, Dijon, France

**Keywords:** meta-omics, microbial indicators, food value chain, biomonitoring, soil quality

## Abstract

Soils are fundamental resources for agricultural production and play an essential role in food security. They represent the keystone of the food value chain because they harbor a large fraction of biodiversity—the backbone of the regulation of ecosystem services and “soil health” maintenance. In the face of the numerous causes of soil degradation such as unsustainable soil management practices, pollution, waste disposal, or the increasing number of extreme weather events, it has become clear that (i) preserving the soil biodiversity is key to food security, and (ii) biodiversity-based solutions for environmental monitoring have to be developed. Within the soil biodiversity reservoir, microbial diversity including Archaea, Bacteria, Fungi and protists is essential for ecosystem functioning and resilience. Microbial communities are also sensitive to various environmental drivers and to management practices; as a result, they are ideal candidates for monitoring soil quality assessment. The emergence of meta-omics approaches based on recent advances in high-throughput sequencing and bioinformatics has remarkably improved our ability to characterize microbial diversity and its potential functions. This revolution has substantially filled the knowledge gap about soil microbial diversity regulation and ecology, but also provided new and robust indicators of agricultural soil quality. We reviewed how meta-omics approaches replaced traditional methods and allowed developing modern microbial indicators of the soil biological quality. Each meta-omics approach is described in its general principles, methodologies, specificities, strengths and drawbacks, and illustrated with concrete applications for soil monitoring. The development of metabarcoding approaches in the last 20 years has led to a collection of microbial indicators that are now operational and available for the farming sector. Our review shows that despite the recent huge advances, some meta-omics approaches (e.g., metatranscriptomics or meta-proteomics) still need developments to be operational for environmental bio-monitoring. As regards prospects, we outline the importance of building up repositories of soil quality indicators. These are essential for objective and robust diagnosis, to help actors and stakeholders improve soil management, with a view to or to contribute to combining the food and environmental quality of next-generation farming systems in the context of the agroecological transition.

## Link Between Soil as a Support for Food Production and a Habitat for Living Organisms

**Figure 1:**
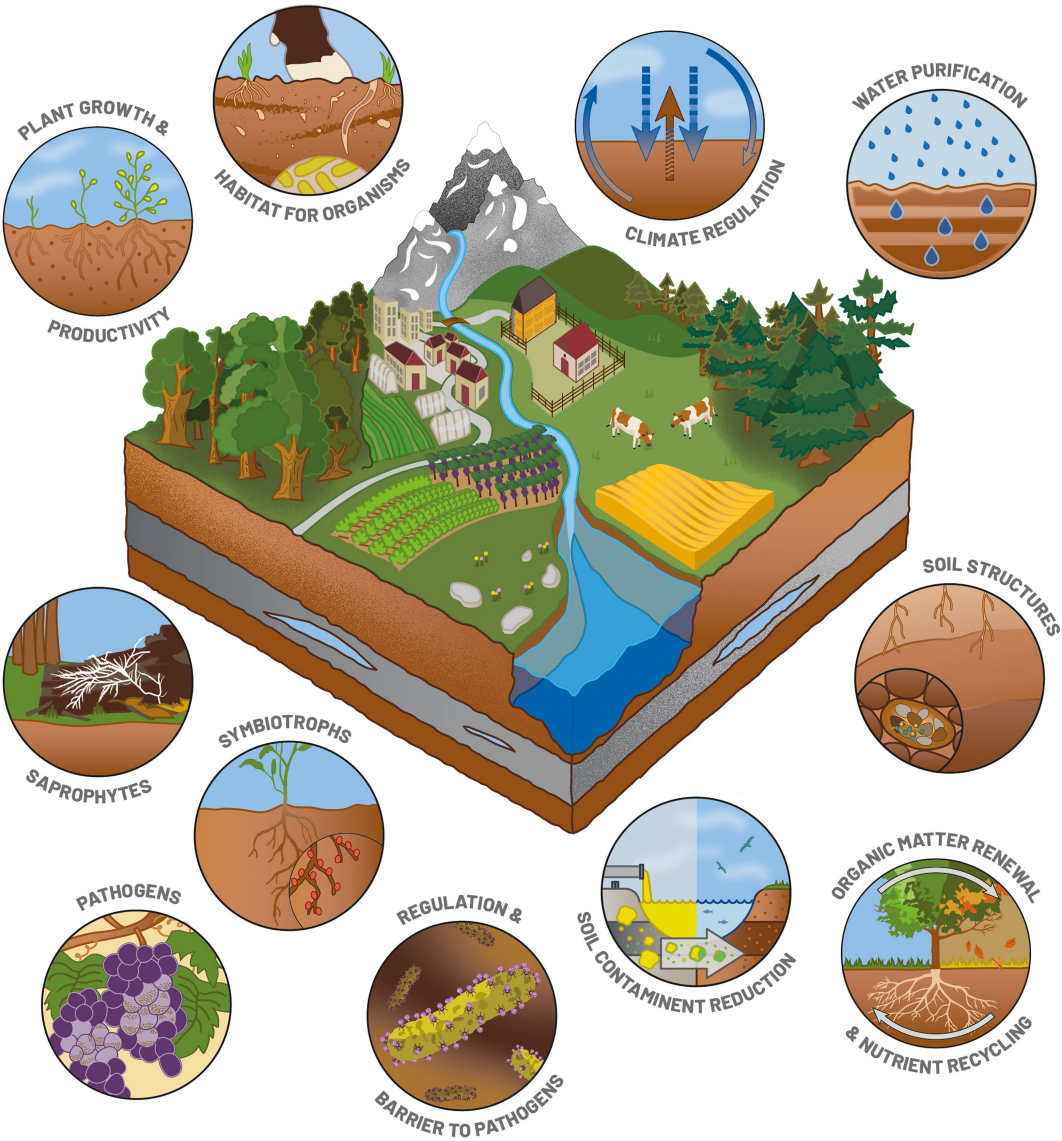
Illustration of the main roles of soil functions and microorganisms as essential players in various ecosystem services.

Soil is the most complex natural physicochemical matrix and the most structured one on our planet. This heterogeneous layer is the most superficial one of the Earth’s crusts, and can be from a few centimeters to several tens of meters deep. The variability of this matrix can be structured into three dimensions: horizontal, vertical, and temporal. Horizontal variability results from the spatial distribution of the different types of soils, influenced by the type of parent material (limestone, granite, metamorphic rock) on a large scale (Chesworth, 2007). Vertical variability refers to the different layers – called “horizons” – that have differentiated over time (Hartemink et al., 2020). Temporal variability results from the pedogenesis process, which is at the origin of soil creation from the weathering of the parent material by climate, vegetation and living organisms or human interventions. Soil formation is a slow process: 0.05 mm of soil are formed each year on average (Evans et al., 2019). Altogether, this makes soils a mosaic of extremely rich and diversified habitats providing a large number of macro- and micro-environments for micro-, meso- and macroscopic living organisms (Nielsen et al., 2015). It is home to many organisms that spend part or all of their life cycle underground and are part of the soil biota (e.g., nematodes, tardigrades, earthworms, microorganisms; Figure 1; Bardgett and Van Der Putten, 2014). Thanks to this mosaic of micro- and macro-habitats, soils host almost one third of our planet’s biodiversity (FAOSTAT, 2021).

The food value chain summarizes the expression “from farm to fork,” including all steps from food production to food distribution to consumers, *via* food manufacturing. Soils are fundamental resources for agricultural production and play an essential role for food security (Kopittke et al., 2022). As providers of more than 95% of food and feed, they represent the cornerstone of the food value chain (Friedrichsen et al., 2019; FAOSTAT, 2021). Soil nutrient availability is unevenly distributed on regional and global scales and affects agricultural productivity (Silver et al., 2021). Soils harbor a large fraction of biodiversity – the backbone of the regulation of ecosystem services and soil multi-functionality. However, these are increasingly threatened by anthropogenic activities such as unsustainable soil management practices, pollution, waste disposal, or the increasing number of extreme weather events. Thus, monitoring the soil biodiversity has become an imperious and urgent issue to secure food production, and requires the development of microbial indicators (Kopittke et al., 2019). New initiatives have recently been launched at a global scale to try and build indicators of food system sustainability (Béné et al., 2019), but microbial diversity was missing from the 27 selected indicators.

### Soil Microorganisms: Modern Bioindicators of Soil Quality in a Context of Agroecological Transition

#### Definition of Bioindicators

A bioindicator (or biological indicator) is a measurement of macro- or micro-organisms whose response in terms of presence/absence, abundance, activities/functions, morphology, physiology or behavior provides information on the state of a habitat or ecosystem (Martinez-Salgado et al., 2010; Astudillo-García et al., 2019). It is used to assess the quality of an environment and its evolution over time. Bio-monitoring of soil quality is carried out at different spatial (local, landscape, regional or global) and temporal (years, decades, millennia) scales, but also at different levels of biological organization (individual, population, community, ecosystem; Pulleman et al., 2012).

Soil bioindicators are used to monitor soil responses to disturbances such as land use changes, agricultural management, or soil contamination, and to assess environmental risks (Geisen et al., 2019). It is essential for a bioindicator to have a good sensitivity to soil management and environmental pressures, and to be relevant to soil functions (e.g., organic matter decomposition, mineralization of soil nitrogen, formation of the soil structure) or related to soil quality (Bloem et al., 2005). From an operational perspective, an indicator should (i) be user-friendly in the field (ii) be precise and reproducible, and (iii) have an affordable financial cost to be used by the greatest number of actors and on a large scale (Griffiths et al., 2018). It should be as universal as possible for the various microbiomes (Schloter et al., 2018). Macro-fauna organisms (e.g., nematodes and earthworms) long remained the only indicators of soil biological quality because they were easily observed and well established in ISO standards (Martinez-Salgado et al., 2010; Schloter et al., 2018; Phillips et al., 2021). Microorganisms were used as bioindicators of soil quality later, but only when the molecular ecology era began (Maron et al., 2011). There are currently several bioindicators of soil quality available, based on microbial taxa and their associated functions (Schloter et al., 2018; Thiele-Bruhn et al., 2020).

#### Microorganisms Are Good Bioindicators of Soil Quality

**Figure 2:**
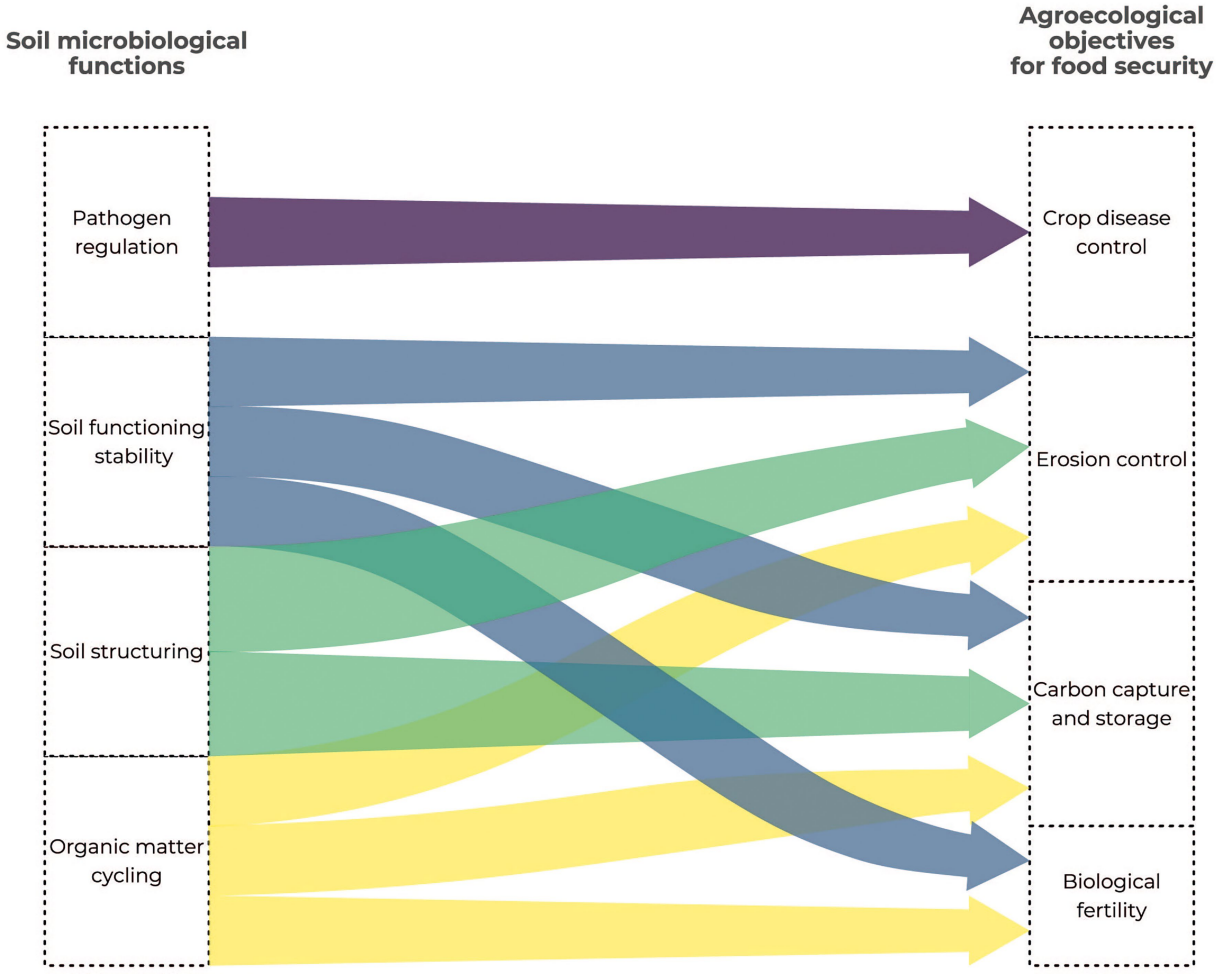
Link between the soil microbiological functions and the agroecological objectives for food security.

Microorganisms (mainly bacteria, fungi and protists) and their functional guilds (pathotrophs, saprotrophs, symbiotrophs) are involved in many ecosystem services (Figure 1), in particular through their role in the biogeochemical cycles of major elements (Hermans et al., 2017; Frac et al., 2018; Geisen et al., 2018). They are involved in N cycling by fixing atmospheric N, denitrification and ammonification (Hayatsu et al., 2008). Soil organic matter mineralization is mainly driven by microbial communities that transform the complex carbohydrates into mineral elements available for plant growth (Nielsen et al., 2015; Maron et al., 2018; Yang et al., 2018). Various microorganisms – especially bacteria and fungi – can achieve symbiosis with plants and benefit to plant growth and productivity (Prudent et al., 2020; Figure 1). They can also control plant pathogens thanks to metabolites and provide a barrier against pathogens (Figure 1). Soil microbial communities deliver other kinds of services that promote the soil structure or reduce soil contamination (FAO, ITPS, GSBI, and SCBD, 2020; Figure 1). Their deep involvement in ecological services make microorganisms ideal candidate bioindicators for monitoring the soil quality and food safety (Figure 2). In addition, their high sensitivity to environmental disturbances combined with their short generation time makes them early indicators of disturbances. As the cost of some meta-omics technologies is getting lower, the trends are now to define news bioindicators, and an inventory of operational bioindicators is necessary to provide a benchmark for soil quality assessment. This evaluation needs to include (i) the currently available meta-omics approaches so as to evaluate their benefits and technical or conceptual drawbacks (ii) the establishment of an adapted sampling design, and (iii) the development of repositories for an objective interpretation of the results.

## Contribution of -Omics Technologies to the Monitoring of the Soil Microbiological Quality

Over the past two decades, many molecular tools have emerged that have accelerated the exploration of the soil microbial diversity, and later contributed to soil quality assessment (Schloter et al., 2018; Nkongolo and Narendrula-Kotha, 2020). These methods can be divided into three major categories: (i) quantitative methods (ii) hybridization methods (microarrays), and (iii) sequencing methods.

### Quantitative Methods

Different quantitative methods have been developed to investigate the relative or absolute abundance of taxa but also their functional profiles by targeting specific genes or gene families of interest (e.g., antibiotic resistome, functional genes in the N cycle; Oshiki et al., 2018). Quantitative polymerase chain reaction (qPCR), high-throughput quantitative PCR (HT-qPCR) and droplet digital PCR (ddPCR) provide access to the numbers of copies of one or more genes. Technological advances have made it possible to simultaneously analyze several thousands of samples and targets (Porter and Hajibabaei, 2018; Mehle and Dreo, 2019). More sensitive and more accurate, ddPCR appears to be promising for an absolute quantification of genes from complex samples such as soil samples because it can overcome the limitations associated with the presence of inhibitors (Gutiérrez-Aguirre et al., 2015).

### Microarray Methods

DNA microarray is based on hybridization between (i) specific oligonucleotides used as probes and fixed on a solid surface, and (ii) the target corresponding to the DNA/RNA soil sample (Srivastava et al., 2019). Combining hybridization and labeling (e.g., by fluorescence) of DNA, this semi-quantitative technology is used to detect the presence or relative abundance of target markers or to monitor gene expression profiles at the individual or community levels (Sessitsch et al., 2006; Porter and Hajibabaei, 2018). Various microarrays such as PhyloChip, GeoChip (He et al., 2007), PathoChip (Lee et al., 2013), StressChip (Zhou et al., 2013), CAZyChip (Abot et al., 2016) have been developed to capture a snapshot of specific microbial populations and/or functional genes. For example, GeoChip was designed to detect genes from various metabolic pathways including the carbon, nitrogen, sulfur and phosphorus cycles. The latest version includes about 160,000 distinct probes covering about 1,500 functional gene families. PathoChip is less widespread and contains over 3,000 probes characterizing virulence factors (e.g., adhesion, colonization, motility, toxins) covering 1,400 species. CAZyChip was developed in 2016 to monitor the expression profiles of genes encoding bacterial enzymes specifically involved in polysaccharide degradation. These DNA chips contain 55,220 probes targeting glycoside hydrolases covering more than 70% of the families present in the CAZy database. Some of these microarrays have become tools for diagnosis, in particular for targeting viruses, bacterial or fungal pathogens or harmful organisms.

### From DNA Fingerprinting to High-Throughput Sequencing: Characterizing Microbial Diversity and Functions

DNA fingerprinting was the first high-throughput analytical technique used to comprehensively characterize the genetic structure of soil microbial communities in terms of presence, absence or relative abundance. These approaches are frequently based on PCR amplification of a molecular marker, and classified in two groups depending on the polymorphism of the studied sequences. The first group is usually based on the sequence length polymorphism and includes T-RFLP (restriction fragment length polymorphism), ARDRA [amplified rDNA (ribosomal DNA) restriction analysis], or ARISA (automated ribosomal intergenic spacer analysis). The second group relies on the polymorphism in the composition of the nucleotide sequence and involves techniques such as DGGE (denaturing gradient gel electrophoresis) or TGGE (temperature gradient gel electrophoresis; Rincon-Florez et al., 2013). These tools have been used for various soil microbial diagnostics because they are inexpensive and relatively fast (Sessitsch et al., 2006; Rincon-Florez et al., 2013; van Dorst et al., 2014). However, their sensitivity only identifies the dominant groups, and the comparison of samples (inter-gel) remains difficult (Schloter et al., 2018). Moreover, they do not provide any identification of the organisms or measurement of microbial diversity. These fingerprinting tools have been totally replaced by high-throughput sequencing.

**Figure 3:**
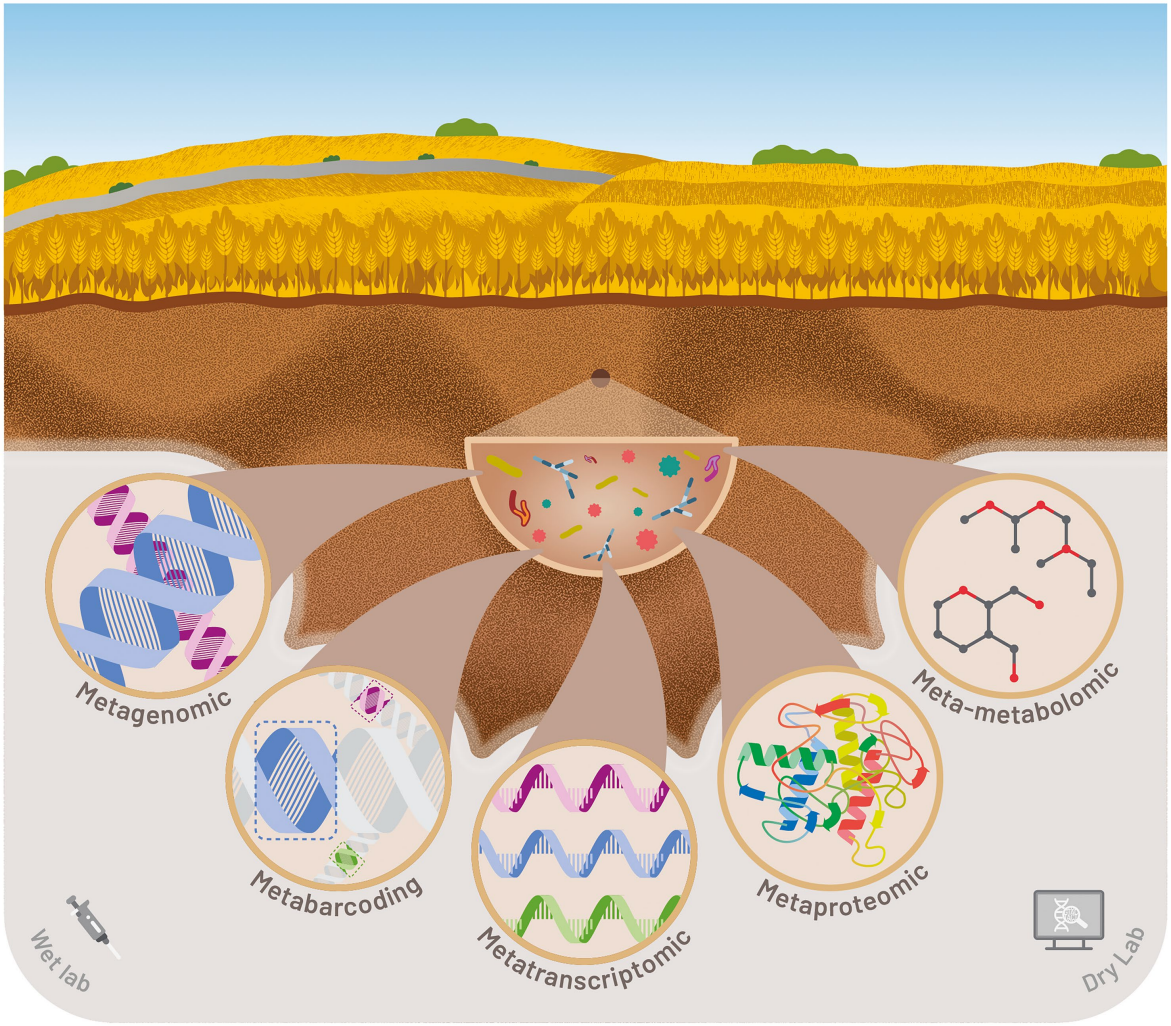
Illustration of various meta-omics approaches for monitoring soil quality and securing food production.

Second- and third-generation high-throughput sequencing offers new ways of answering those various technical and scientific issues. Sequencing coverage, raw data quality, read length, technical biases vary depending on the method (Goodwin et al., 2016). Nevertheless, the performance of high-throughput sequencing platforms is constantly evolving to better support scientific goals at increasingly affordable costs. This has enabled the democratization of several meta-omics approaches for scientific teams but also the transfer of bio-indicators for the agri-food industries. In the next section, we explore and describe the different promising meta-omics approaches for the development of soil quality bioindicators (Figure 3).

## Meta-Omics Approaches: Guidelines for and Applications to the Food Value Chain

**Figure 4:**
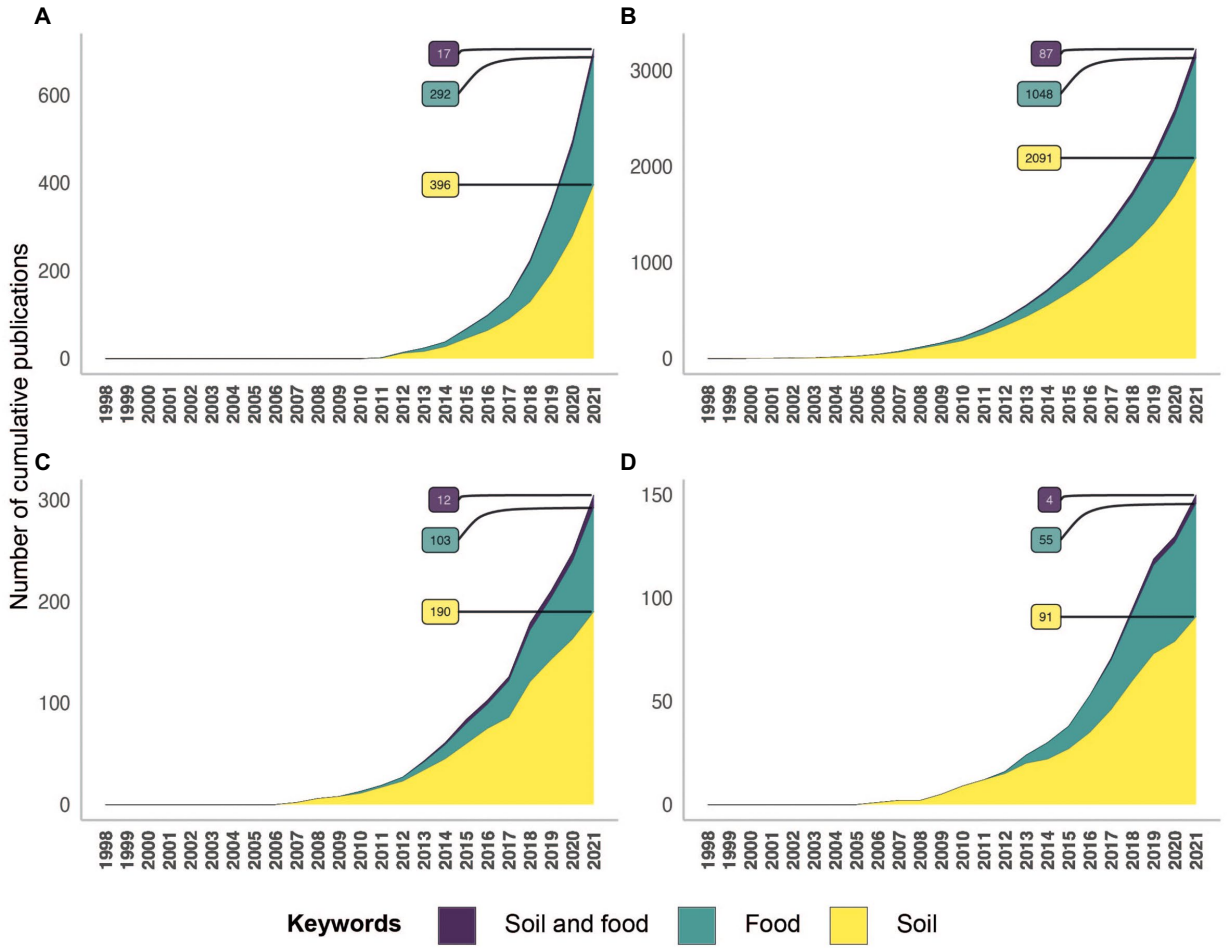
Temporal evolution of the number of references indexed in the Pubmed database concerning meta-omics approaches in relation to soil (yellow), food (green) and soil and food (purple). This analysis relies on keywords present in the title or abstract, related to meta-omics (metabarcoding **(A)**, metagenomics **(B)**, metatranscriptomics **(C)** and meta-proteomics **(D)**) across the different research topics (soil; food; soil and food). Concerning the analysis of metagenomics keywords, these can be biased because a lot of publications used “metagenomics” instead of metabarcoding.

**Figure 5:**
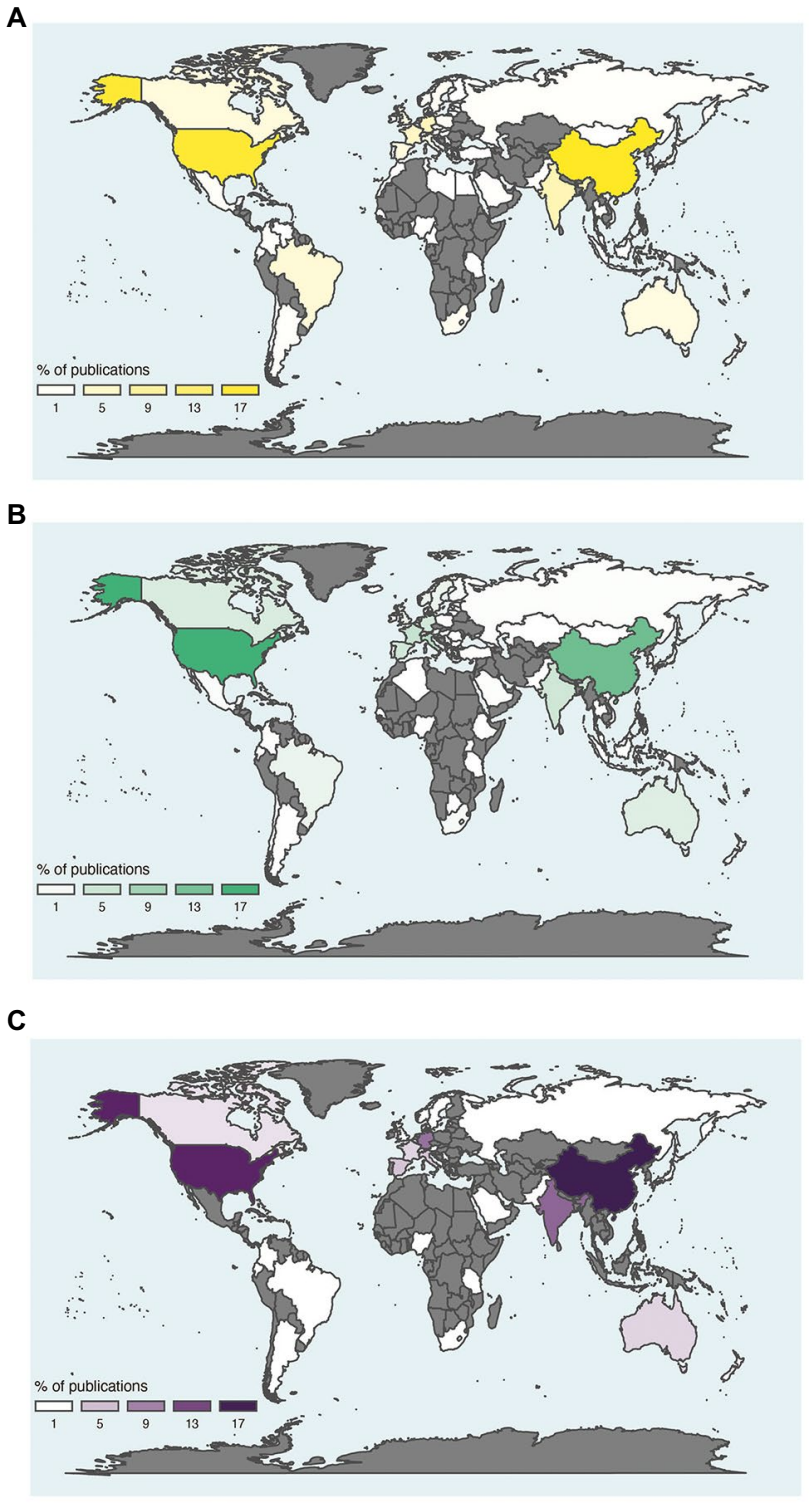
Distribution of publications on meta-omics approaches in relation to soil **(A)**, food **(B)** and soil and food **(C)**. World distribution of publications on soil **(A)**, food **(B)** and soil and food **(C)** using meta-omics approaches. This analysis relies on keywords present in the title or abstract, related to meta-omics (merging studies on metabarcoding, metagenomics, metatranscriptomics and meta-proteomics) and based on the country of the first author. Gray areas, absence of publication.

A paradigm shift emerged in environmental microbiology studies at the end of the 1990’s, as the studies based on culture-dependent methods disappeared and were replaced by culture-independent methods. The transition was facilitated by our capacity to extract and access the molecular resources (DNA, RNA, proteins, metabolites) of microbial communities directly from environmental samples. This determined the beginning of the present meta-omics area. These high-throughput approaches for screening hundreds to thousands of samples simultaneously and retrieving a huge amount of data have been developed and have evolved considerably over time. Studies related to soil and/or food have known rapid and important progress thanks to meta-omics since 2010 (Figure 4). However, very few studies related to soil and food were based on meta-omics compared to the number of studies focused on soil or food independently of each other. The metagenomic and metabarcoding surveys were the most frequently used approaches to study the soil and food microbiota and the microbiome, while metatranscriptomics and meta-proteomics were rarely investigated (Figure 4). The countries that produced the greatest numbers of publications about soil and/or food microbial research based on meta-omics were the USA and China, followed by Germany and France for European countries (Figure 5).

### Assessing Analytical Specificity in Soil Analysis

#### Consequences of the Sampling Effort and Sample Conservation on Soil Analysis

Any sampling survey first involves a series of decisions that determine how the data should be analyzed (Dickie et al., 2018), e.g., what is sampled, what is not sampled, where it is sampled, how many samples to be taken, how many replicates, location of subsamples within plots, etc. Even storage conditions of soil samples can influence the quality of the molecular resource and should be well adapted. For example, the use of sieved soils and − 40°C storage is efficient to access and keep DNA, but not to preserve and extract a more sensitive molecule like RNA (Ranjard et al., 2009). Fresh soils are undoubtedly an ideal material for characterizing microbial taxonomic and functional diversity, but using them is sometimes impossible in the case of large sampling surveys or comparisons among chronological samples requiring storage conditions (Cui et al., 2014; Table 1).

**Table 1:** Influence of the conservation strategy of the soil sample on the quality of the extracted biological material.

**Preservation strategy**	DNA	RNA	Proteins	Metabolites
Immediate sample processing	+++	+++	+++	+++
Drying (room temperature)	+/−	−	−	−
Cold storage (4°C)	+/−	−	−	−
Freezing (−20°C)	++	+/−	+/−	+
Low freezing (−40°C to −80°C)	+++	+++	+++	+++
Chemical preservation (use of stabilizing solution)	+/−	++	++	unknown

*+++, Image close to that of the sample; ++, Good protection of the sample; +, Average protection of the sample; +/−, Moderate impact of the storage method on the image; −, Strong impact of the storage method on the results.*

#### Extraction of Soil Genetic Resources: DNA and RNA

Meta-omics approaches started with isolating DNA directly from environmental samples. Success essentially depended upon the quality and the quantity of isolated DNA (Terrat et al., 2015). Various homemade DNA extraction protocols and commercial kits were developed to improve the efficiency of soil DNA extraction (Martin-Laurent et al., 2001; Delmont et al., 2011; Terrat et al., 2012). However, each method is characterized by its own advantages and biases, leading to variations in DNA representativeness. These variations have consequences on the observed effects on soil microbial assessments, and this makes it difficult to compare studies.

The choice of a specific DNA extraction method determines the inherent bias involved throughout the whole project (Vestergaard et al., 2017). This is why standardized methods (like the ISO 11063: 2012 standard) are advised in order to provide consistent results across studies, more particularly when measurements are interpreted relatively to references used as bioindicators of soil quality (Table 1).

DNA provides information only about the presence of microorganisms in soils but does not inform on microbial activities. On the contrary, RNA analyses potentially describe what those microorganisms are actually doing and how fast they are doing it (Prosser, 2015). But RNA is a very sensitive molecule, with a low stability and a short half-life time (Hambraeus et al., 2003), and needs to be protected immediately after soil sampling using dedicated methods or solutions (e.g., LifeGuard Soil Preservation Solution or RNA*later*). Moreover, to protect RNA from degradation by RNases, a clean laboratory must be dedicated. Inactivation reagents for RNases such as guanidine thiocyanate must be added to extraction buffers to inactivate RNase molecules immediately after their release from lyzed cells. Finally, no method for RNA extraction from all types of soils is available; this hinders the study of microbial gene expression in soils (Wang et al., 2012).

#### Extraction of Soil Molecular Resources: Proteins and Metabolites

Due to its huge potential for linking functional and phylogenetic information on soil microbial communities, research in soil ecology has showed a rising interest in meta-proteomics to investigate microbe-driven ecosystem functions (Prosser, 2015; Keiblinger et al., 2016). However, the application of meta-proteomics to soils still faces several challenges such as the heterogeneity of soil matrices or the ecosystem-specific dominance of a few microbial species, which limit the development of a generic protocol. The extraction of soil proteins is sensitive to the presence of several organic compounds such as complex carbohydrates, lipids, phenolics (e.g., lignin), humic substances, but also inorganic compounds such as silt and clay minerals (Keiblinger et al., 2016). Proteins may be adsorbed or linked anchored or embedded onto solid particles and soil organic matter, hence reduced extraction efficiency. In addition, measurements of soil enzyme activities can be affected in different ways depending on the storage method, the soil type and even on the assay method. Freezing of small sampled aliquots retains the activity of most enzymes, but ultra-freezing is preferred for storage of samples with a high organic matter content (Wallenius et al., 2010). Altogether, processing fresh samples whenever possible or storage at −80°C is recommended to minimize the activity of naturally occurring proteases in environmental samples (Keiblinger et al., 2016).

Soil organic matter – more precisely the dissolved fraction – is composed of many small molecules of plant and microbial origin (Swenson et al., 2015). Classical methods for extracting soil metabolites involve long extraction times followed by compound-specific analyses (Swenson et al., 2015). However, the soil physical properties such as mineral composition, surface area, shape and porosity represent different challenges when it comes to extracting soil metabolites and metabolomics approaches. For example, amines adsorb to clay material in soils, and so do other metabolites, especially carboxylic acids that potentially form covalent bonds with organic matter or are chelated by metals or other ions (from the soil or from the extractant). Moreover, each metabolite has specific half-life times depending on the soil specificities, but also on the activities of the organisms (i.e., enzymatic degradation). Finally, the quantity, stability and specificity of metabolites after extraction can be influenced by soil storage conditions (Table 1).

### Metabarcoding to Investigate Microbial Diversity

Metabarcoding, also known as targeted metagenomics, uses the polymerase chain reaction (PCR) method to target a genomic region common to all studied microorganisms (Semenov, 2021). This molecular marker can be a gene allowing to study the relationships between microorganisms (considered as a taxonomic marker; Woese et al., 1990) or a functional gene/family (considered as a functional marker; Barbi et al., 2014). DNA fragments (amplicons) produced by the PCR and sequenced by high-throughput sequencing provide an overview of microbial diversity (Nilsson et al., 2019a). Several molecular markers exist to characterize different microbial communities. The most popular marker is the gene encoding the small subunit ribosomal RNA (SSU rRNA), essential to ribosome formation. The 16S rRNA gene is used to determine the diversity of Archaea and Bacteria (Woese and Fox, 1977). Similar to the 16S rRNA gene, the 18S rRNA gene is used to explore eukaryotic organisms, and in particular the diversity of the fungal kingdom (Hadziavdic et al., 2014). Considered as the universal DNA barcode marker to investigate fungal diversity, the nuclear ribosomal internal transcribed spacer (ITS) is also applied to identity fungi. Among further taxonomic markers of other important functions are the *gyrB* (DNA gyrase subunit B), *recA* (recombinase A), or *rpoB* (RNA polymerase subunit beta) genes (Holmes et al., 2004; Poirier et al., 2018; Ogier et al., 2019). The choice of the marker depends on the targeted scientific question/objective and microbial communities. Using the metabarcoding approach is advantageous in several ways. It has become very popular in the last decade, and is undoubtedly the most affordable meta-omics approach. Thus, many projects have emerged to evaluate soil quality at large and local scales. Data analysis is now fully operational thanks to a significant range of open-source bioinformatics pipelines such as mothur (Schloss et al., 2009), qiime2 (Bolyen et al., 2019), FROGS (Escudié et al., 2018), or BIOCOM-PIPE (Djemiel et al., 2020a) and the associated standard operating procedures (SOPs) or tutorials. Nevertheless, this wide choice requires grasping well the subtleties of the algorithms implemented in the pipelines and their parameters because the accuracy of results is highly dependent on them (Pauvert et al., 2019). Another major effort in this area is the availability of reference databases for taxonomic assignment whatever the molecular marker (Quast et al., 2013; Nilsson et al., 2019b; Djemiel et al., 2020b). It should also be emphasized that some sequences could contain misidentifications or errors (Hofstetter et al., 2019). Therefore, it is important to be careful when interpreting taxonomic results, especially at the genus or species ranks. The initiation of a metabarcoding project requires choosing a molecular marker and then one or more regions to be amplified. This choice is crucial because the regions do not all have the same resolution and depend on the targeted samples and communities (Banos et al., 2018; Bukin et al., 2019). As a result, amplicon length is also crucial and impacts on the sequencers to be chosen and library preparation (Nilsson et al., 2019a; Castaño et al., 2020). The sequence length generally ranges from a few dozen to one hundred base pairs; this size does not allow fine taxonomic resolution to determine the species names.

The use of metabarcoding approaches to monitor soil quality and also other compartments of the food chain value is scarce. However, some studies have already applied such approaches and brought significant knowledge on microbiota interactions (Stefanini and Cavalieri, 2018). In a recent study, Zarraonaindia and colleagues investigated 725 samples from soils and grapevine organs using a metabarcoding approach to determine the influence of the vine cultivar., edaphic parameters, the vine developmental stage (dormancy, flowering, pre-harvest), and vineyard characteristics on the spatial and temporal dynamics of bacterial communities (Zarraonaindia et al., 2015). They concluded that most organ-associated taxa originated from the soil, and their distribution reflected the influence of highly localized biogeographic factors and vineyard management. Moreover, the vineyard-associated localization of bacterial taxa has implications for wine growers who rely on the assumption that the soil imparts a unique quality to the wine specific to each vineyard, called the “terroir effect.” Complementary to the evaluation of a microbial terroir, such studies investigate the environmental and human-related factors that influence the composition of microbial populations and potentially affect their performances, like fermentation (Stefanini and Cavalieri, 2018).

### Metagenomics to Explore the Functional Potential of Microbial Communities

The metagenomics approach – or shotgun sequencing (without any *a priori* compared to metabarcoding) – rests upon the sequencing of total DNA from environmental samples. The extracted DNA is directly sequenced (or after fragmentation or nebulization). This avoids PCR amplification, and non-culturable and/or unknown organisms can be characterized, such as viruses (Prosser, 2015). Its main purpose is to characterize whole microbial genomes across microbiome samples. With the increasing efficiency of sequencing technologies, this approach can now be applied with a sufficient amount of acquired sequences to reconstruct “population genomes” of major microorganisms (Prosser, 2015). Metagenomics leads to an overview of the potential genomic structure of the microbial community, its potential functional properties, in a more sensitive manner than metabarcoding does (Bünemann et al., 2018). Moreover, with the democratization of sequencing technologies, a variety of tools and analysis pipelines have been developed to analyze metagenomics data (Kieser et al., 2020). Configuring various tools, linking them with advanced binning and annotation tools, and managing the updates and parameters modifications of each tool is now easier and more accessible for researchers with the efficient and automated deployment of workflows like ATLAS, MG-RAST or MOCAT2 (Meyer et al., 2008; Kultima et al., 2016; Kieser et al., 2020).

The use of metagenomics for characterizing microbial communities presents several advantages. As already stated, metagenomics characterizes unknown microorganisms in the absence of prior knowledge, and provides information on potential functions based on the genomic content. This allows a deeper understanding of the response of microbial communities to environmental changes (Aguiar-Pulido et al., 2016a). Some drawbacks have also been identified. The presence of a functional gene is not proof of its expression. The host organism may be dormant, inactive or only active when alternatively expressed metabolic pathways do not require the function of the detected gene (Prosser, 2015). Moreover, for economic reasons the resulting datasets often have low coverage of the genomes in the microbiome. Finally, even with available bioinformatics pipelines, the analysis of such datasets needs expertise and can be time-consuming (Aguiar-Pulido et al., 2016a), with a high percentage of reads considered as “Unknown” due to the absence of similar reference genomes.

Metagenomics outcompetes metabarcoding in that it provides insights into the metabolic, virulence or resistance potential of microbial communities. A good example is the study of Wind and colleagues (Wind et al., 2021) focused on antibiotic resistance genes (ARG) across a vegetable production system. The study was interested on three pre-harvest “control-points,” i.e., manure and compost amendments, soils, and lettuce samples, to track antibiotic resistance and the associated microbiomes in order to identify the potential impacts of key agricultural management strategies. The soils were found highly stable in the composition of the resistome, which has the potential to act as a natural ecological buffer to ARG proliferation, at least over one harvest cycle. But lettuce grown in soils amended with compost produced from the manure of cows had higher total relative ARG abundance and risk scores compared to the soil amended with stockpiled manure. Similar results were described with poultry manure application: ARG abundance increased on lettuce surfaces at the greenhouse scale (Zhang et al., 2019). The authors of these studies advise reducing the spreading of ARG from amendment onto the lettuce leaf surface as this may influence human health negatively. Many other scientific questions can be tackled using metagenomics, such as Zn availability in grains for biofortification (Wang et al., 2021), or the detection of foodborne pathogens in production chains (Yang et al., 2016).

### Metatranscriptomics Dedicated to Studying Gene Expression in Microbial Communities

Metatranscriptomics is quite similar to metagenomics, but different in that it focuses on microbial gene expression from complex environments. Metatranscriptomics provides the diversity of the active genes within a community, and measures the changes in the level of expression due to modified environmental conditions (Mukherjee and Reddy, 2020). Extracted RNAs are reverse-transcribed into cDNA and sequenced, as in metagenomics studies. Pioneering studies aiming to identify expressed genes in environmental samples date back to 2005 and represent the dawn of metatranscriptomics (Prosser, 2015). As in metagenomics studies, reads are either mapped to a reference genome or *de novo* assembled into contigs and scaffolds (Prosser, 2015). Several advantages of metatranscriptomics can be highlighted. This approach characterizes unknown microorganisms, as metagenomics does. It is complementary to metagenomics in that it provides a snapshot of expressed genes (as only coding sequences are obtained). Thus, the active microbiome is explored to understand how patterns of gene expression change following biotic and abiotic stresses and various environmental disturbances. Yet, although it is easy to recover large quantities of total RNA from a pure bacterial culture (tens of micrograms *per* extraction), this is rather difficult in soil samples (Wang et al., 2012). According to reports, the extraction yields of soil RNA range from tens of nanograms to several micrograms *per* gram of soil. Such a range could be explained by the amount of living microorganisms in the soil samples, by contamination by humic substances or by RNA loss during purification (Wang et al., 2012). The technical issues associated with metatranscriptomics are similar to those associated with metagenomics, but with further potential biases introduced during rRNA depletion (as rRNAs can represent more than 80% of total RNAs), the construction of cDNA libraries, and the required rapid inactivation of samples to prevent mRNA turnover (Prosser, 2015).

As metatranscriptomics approaches can be complicated to apply across the whole food value chain, only specific compartments have been studied. For example, the microbiota of various Swiss-type cheeses were analyzed during ripening (Duru et al., 2018). Samples were collected from three cheeses at two time points – during warm room ripening and cold room ripening. The resulting data supported a better understanding of the flavor-forming mechanism, i.e., the succession of up-regulated and down-regulated pathways involving microbial species during warm and cold ripening of the cheese. Such studies are a first step toward improving the use of food microbiomes in terms of flavor, quality and security (Afshari et al., 2020).

### Characterization of Microbial Community Functionality and Activity: Meta-Proteomics and Meta-Metabolomics

Meta-proteomics and meta-metabolomics are devoted to the description of microbial functionality and activity. Therefore, they are the functional complement of studies focused on DNA and/or RNA. Extracellular enzymes synthesized by microbial communities contribute to various ecosystem services such as organic matter decomposition or nutrient cycling (Van Emon, 2016). For this purpose, meta-proteomics characterizes the entire protein content of an environmental microbiome at a given location and time. Meta-metabolomics is dedicated to the comprehensive analysis of all metabolites (i.e., small molecules released by microorganisms into the environment) contained in a sample. Due to its large potential for providing a link between functional and phylogenetic information on soil microbial communities, there has been growing interest in the application of meta-proteomics in soil ecology to study microbe-driven ecosystem functions (Keiblinger et al., 2016). Bacteria, fungi and protists indeed excrete a wide range of volatile organic compounds to interact and communicate with each other, promote crop growth or modulate plant defense (Schulz-Bohm et al., 2017; Poveda, 2021).

Both approaches use common methodologies to extract proteins or metabolites from soil matrices. Direct extraction can be applied, using physical, chemical or mechanical lysis of cells, leading to comprehensive protein (or metabolite) recovery from soil microorganisms. For the choice of the cell lysis method, the target proteins and soil texture should be considered (Keiblinger et al., 2016). This direct approach is often complex due to the presence of other organic compounds co-extracted with proteins or metabolites. These co-extracted products can also complexify the analysis steps. Moreover, the choice of the extraction method can clearly impact the results of the meta-metabolomics approach. Another solution consists in separating microbial cells from the soil matrix prior to extraction, e.g., by density gradient centrifugation (Keiblinger et al., 2016). This indirect extraction reduces the problems caused by interfering and coextracted substances. However, all proteins, enzymes and metabolites excreted by microorganisms are ignored.

Generating meta-proteomics and meta-metabolomics data differs significantly from generating metagenomics and metatranscriptomics data, which rely heavily on sequencing (Speda et al., 2017). Concerning meta-proteomics, the complexity of environmental samples still outstrips the capabilities of mass spectrometry approaches (Keiblinger et al., 2016). Thus, protein separation is mandatory to reduce sample complexity before mass spectrometry analysis. 2D gel-based protein separation methods prior to enzymatic digestion can be successfully employed, but these methods have major drawbacks, particularly regarding the analysis of proteins with extreme molecular weights, isoelectric points or hydrophobicity values. Gel-free approaches include different protein extraction procedures, followed by digestion into peptides. Peptides are further separated by reversed-phase liquid chromatography or 2D chromatographic separation using strong cation exchange chromatography in combination with reversed-phase liquid chromatography. Then, peptides are analyzed by mass spectrometry, the measured spectra are compared with theoretical spectra of peptides from a database. Due to its high efficiency and automation, this method is the main strategy for protein identification, quantification and detection. However, only protein sequences represented in the database can be identified.

Identifying and quantifying metabolites is typically carried out using a combination of chromatography techniques (i.e., liquid chromatography, gas chromatography) and detection methods, such as mass spectrometry, and nuclear magnetic resonance (Aguiar-Pulido et al., 2016b). These technologies produce spectra consisting of patterns of peaks that allow metabolite identification and quantification, using comparisons with spectral databases like BioMagResBank, the MassBank, or the Golm Metabolome Database (Aguiar-Pulido et al., 2016b). Prior to this analysis, denoising and peak-picking processes are essential to improve the quality of the treated data.

Data storage and management are a major issue for both approaches because the generated data take valuable space and are barely standardized (Keiblinger et al., 2016; Aguiar-Pulido et al., 2016b). Moreover, meta-proteomics and meta-metabolomics are relatively costly in terms of equipment, and need a high degree of scientific knowledge to compute, analyze and treat the data. But, contrary to other meta-omics approaches, meta-proteomics and meta-metabolomics are the most direct methods for identifying the functionalities of a natural environment because they work as semi-quantitative methods. Moreover, as these approaches are very sensitive, they can be considered as a good bioindicator of soil quality. Due to their large potential for providing precise information on soil microbial communities, meta-proteomics and meta-metabolomics are currently most advanced among meta-omics approaches.

In parallel to metatranscriptomics, meta-proteomics and meta-metabolomics are generally applied only for specific compartments of food value chains (soils, plants, or transformed products). For example, the composition of soybean leaf metabolites was investigated using metabolomics (Yun et al., 2018) and linked with the soil physicochemical properties. The authors characterized leaf metabolites of 40 samples harvested at two growth stages and from two contrasting sites, with ten replicates *per* condition. They concluded that the soybean leaf metabolome is clearly dependent on the geographical area and influenced by environmental factors such as the soil properties. However, recent studies are a starting point for further, more detailed studies of more compartments of food chains (Mattarozzi et al., 2020). These authors applied meta-proteomics on rhizosphere soils to have a better understanding of the impact of biostimulants on maize seeds. They demonstrated that biostimulants increased the activity of the bacterial community to different extents, and created a permissive and nurturing rhizosphere by stimulating microbial metabolism. As a result, plant growth and root elongation were promoted, and the activity of the bacterial community – especially of species beneficial to plant growth – was increased, without the composition of the microbiota being modified.

## Contribution of Meta-Omics to Soil Quality Indicators

### Issues and Obstacles to Making Meta-Omics Usable for Operational Bioindicators

As previously detailed, meta-omics can be used to characterize the soil microbial communities and evaluate the soil biological quality, but environmental heterogeneity can complexify the analyses. For microbial communities, environmental heterogeneity provides a multitude of habitats at the microscale and macroscale (Fierer, 2017; Karimi et al., 2020). Moreover, soil physico-chemical differences across fields, landscapes, regions and/or continents create an extraordinary pedodiversity that complexifies the study and the survey of soil microbial communities (Hermans et al., 2019). To ensure robust study strategies and avoid the effects of storage time and conditions on the microbial community composition, several international standardized methods (like ISO standards) have been developed to recommend the most accurate sampling method (composite, stratified, systematic, random) and storage conditions of soil samples.

**Figure 6:**
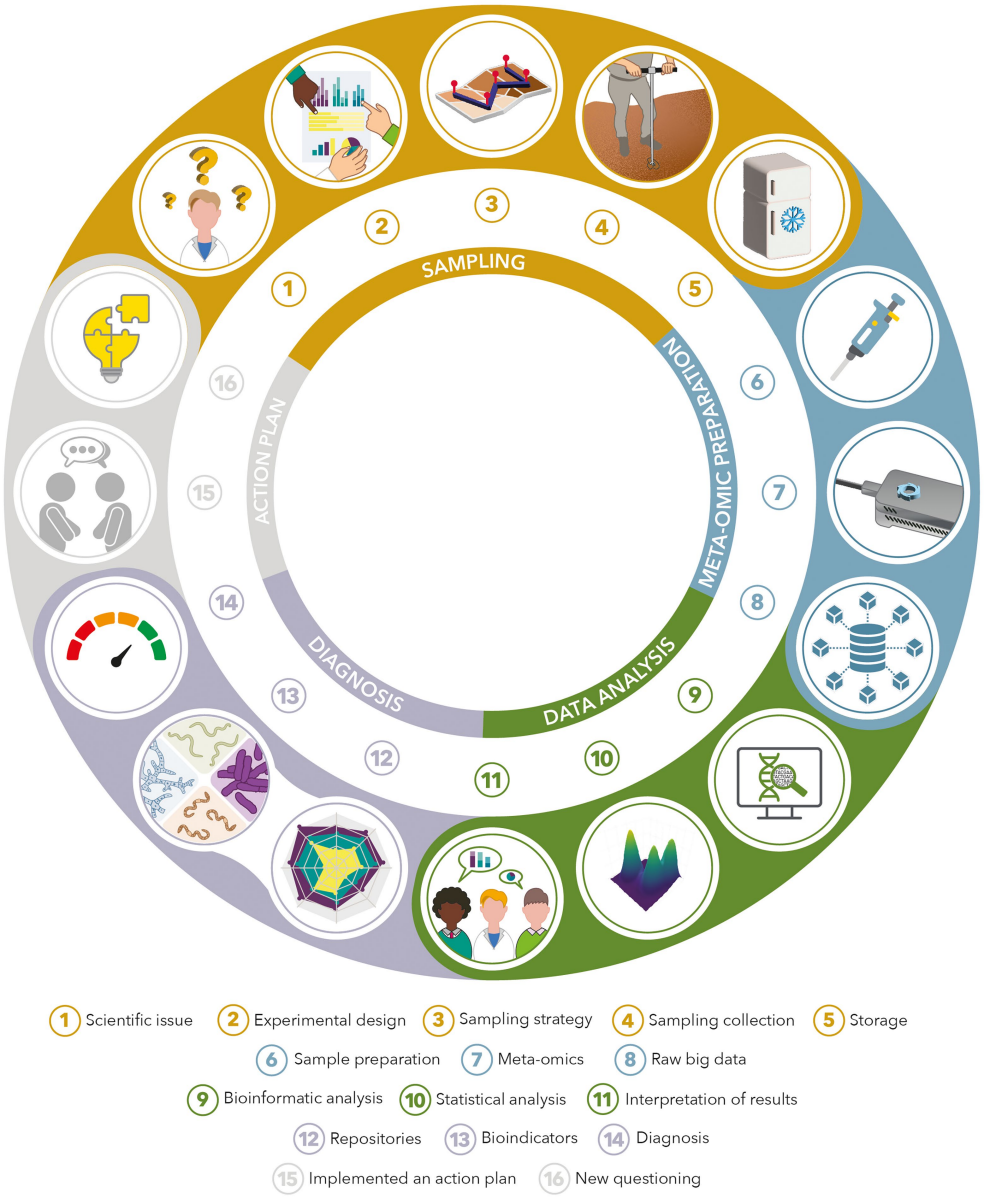
Example of a strategy for studying soil samples with meta-omics approaches: from basic research issues to operational diagnosis.

Along with the application of meta-omics methods to characterize microbial diversity in soil samples, several criteria must be verified to lead to an operational bioindicator (Figure 6). First, the repeatability, sensitivity and reproducibility levels of the method must be informed and controlled. Moreover, standard operating procedures must be created, evaluated, and tested by independent laboratories (Terrat et al., 2015). The chosen meta-omics procedure must be easily usable as routine analysis (Supplementary Table 1).

**Figure 7:**
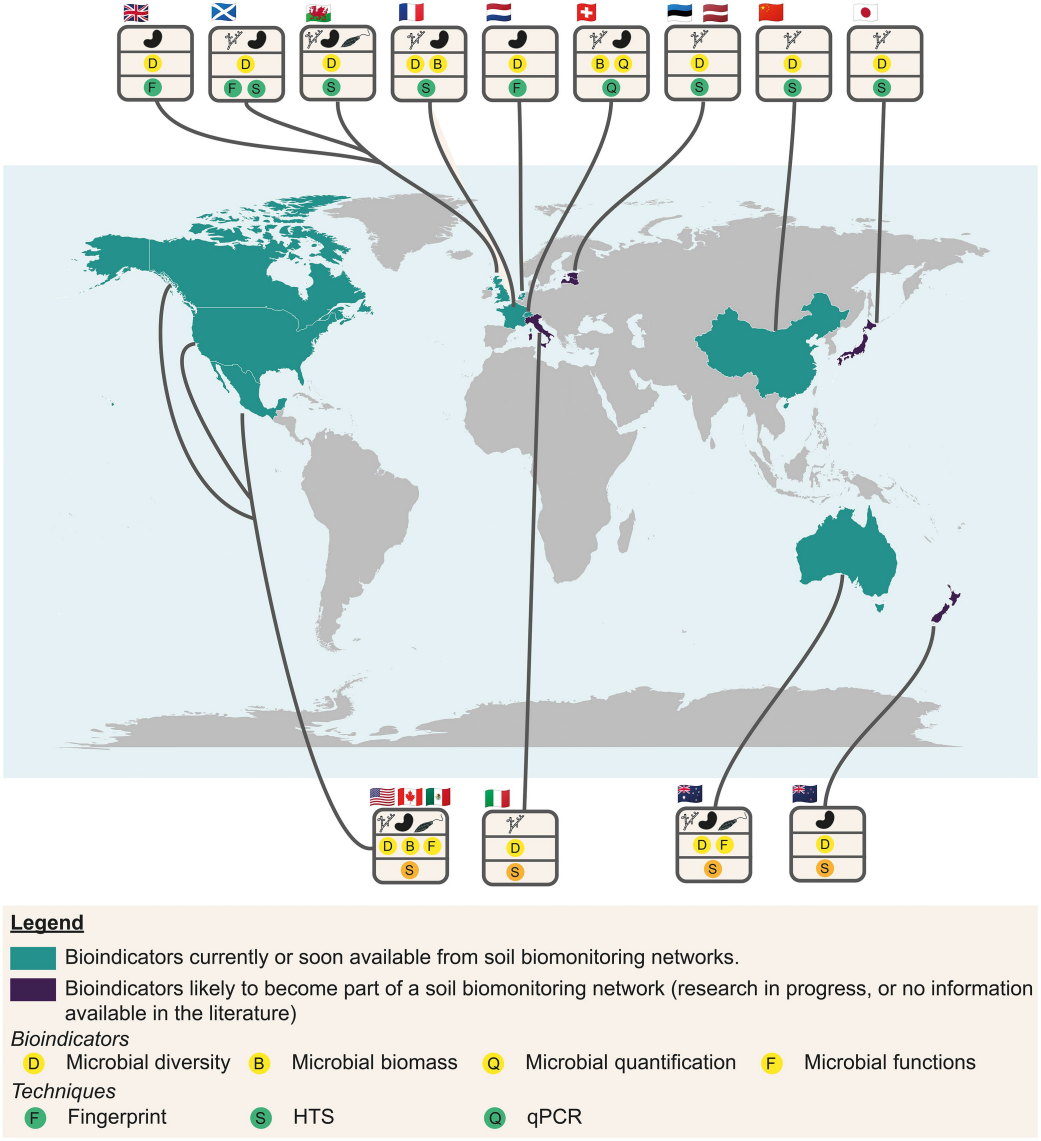
World map showing the main surveys used to monitor the soil quality thanks to repositories based on modern and operational microbial indicators or in progress (turquoise) and studies in the research phase (purple). Microbial indicators are symbolized by circles with a yellow background, and methods by circles with a green background.

The understanding and interpretation of any bioindicator of soil quality needs a repository to diagnose the biological status of soils (Lemanceau et al., 2014; Hermans et al., 2017; Jansson and Hofmockel, 2018). Such repositories are used to evaluate whether the measured values are within the range of variations considered as “normal operating range,” namely for a given soil type and land use. Data rely on prior descriptive biodiversity studies, the determination of the relationships between microbial abundance and diversity, taxonomic abundance, microbial activities and functioning, and environmental filters (soil, physicochemical, meteorological and spatial parameters). This knowledge of microbial biogeography needs studies at a large spatial scale. A significant number of soil microbial surveys are listed at the national or territory scale (Figure 7). Some of these studies aim to monitor soil quality thanks to repositories, and propose modern and operational microbial indicators (Figure 7; Rutgers et al., 2009; Griffiths et al., 2011; Powell et al., 2015; Bissett et al., 2016; Horrigue et al., 2016; Terrat et al., 2017; Tresch et al., 2018; George et al., 2019; Ji et al., 2019; Norris et al., 2020). Some are used to measure the impact of land use changes combined with pedoclimatic conditions (Horrigue et al., 2016; Terrat et al., 2017), or of farming practices (Norris et al., 2020) or to evaluate the quality of urban soils (Tresch et al., 2018). The main measured indicators are microbial biomass, microbial diversity (16S, 18S rDNA and ITS markers) using metabarcoding, and more rarely the microbial functional potential through metagenomics (Bissett et al., 2016; Norris et al., 2020). Other research works have sought to understand the spatial distribution and the main environmental drivers, but can be considered as microbial monitoring frameworks for practical conservation of soils (Toju et al., 2016; Hermans et al., 2017; Coller et al., 2019; Jiao and Lu, 2020; Tedersoo et al., 2020; Figure 7).

### Examples Using Meta-Omics to Assess Soil Quality as a Part of the Food Value Chain

The extensive use of meta-omics approaches to better characterize soil microbial communities has clearly improved our knowledge on soil functioning. However, the integration of -omics data to understand and evaluate all the compartments of the food value chain remain scarce and only some recent studies used it to deal with the microbiological risk assessment or the agroecological transition, with the objective of a high food chain quality.

#### Impact of Agricultural Practices on Food Security

Soil fertilization with animal-based organic products, such as manure, can cause human pathogens to develop in the soil (Berge et al., 2009; Semenov et al., 2009). One of the greatest challenges for microbiologists is the exploitation of -omics data in microbiological risk assessment (Cocolin et al., 2018). To date, few studies using metagenomics have assessed the pathogenic status of amended soils (Fang et al., 2015; Meneghine et al., 2017). The most dominant human pathogens detected in soil after a long-term application of chicken manure would be *Mycobacterium tuberculosis* and *Mycobacterium ulcerans* (Fang et al., 2015). The study of (Meneghine et al., 2017) used whole metagenomics sequencing approaches to investigate the taxonomic and functional profiles of microbial communities in soil amended with organic compost made from animal carcasses. Bacterial pathogens generally affiliated with fecal contamination were found in low abundance in the soil (less than 0.4% of the total identified bacterial genera), and no toxigeny-related gene or fecal contaminant was detected. More recently, virulence genes and ARGs were searched for in long-term manure-amended greenhouse soils using metagenomics (Zhang et al., 2020). Manure application led to the accumulation of such virulence and ARGs in soils, and accumulation increased with the frequency of manure application. Such results highlight a risk of widespread ARGs and virulence genes in amended agricultural soils, that can be regulated by mitigating manure application frequency or by pre-treating manure.

#### From Soil Microbiology to Food Quality at the Territorial Scale

The agroecological transition praised at a global scale, but it operates at local scales where particular environmental, social and economic constraints weigh on agricultural production (Schmitt et al., 2017). Based on this observation, projects are now developing that target focused areas and take into account the whole food value chain, from soils to food quality and consumption, including economic aspects (Magrini et al., 2019).

In France, products with labels of quality of origin (e.g., Protected Designation of Origin, aka. PDO; Protected Geographic Indication; “Label Rouge”) are good examples of food value chains in which the quality or the specificity of the products are related to the specificities of the “terroirs” of production, including the local soil properties. Production specifications have long been established independently of environmental issues, hence potential soil degradation. The question of the relationship between the soil ecological quality and the quality of agricultural products has been regularly suggested in recent years, but it still needs objective scientific demonstrations. The IFEP project was set up to bring new scientific knowledge in this field by focusing on the relationship between soil and product specificity for Comté PDO cheese. It aims at investigating the microbial communities along the continuum between the soil, the grass phyllosphere, cow teats and milk, using a metabarcoding approach and across a network of 44 dairy farms (Chemidlin Prévost-Bouré et al., 2021). The results highlighted strong and significant links between the four compartments of this continuum. These links were modulated by environmental factors: soil pH, plant diversity and elevation, but also farming practices, in particular organic fertilization, cattle density and cow-teat care. Thanks to metabarcoding, the IFEP project provided a proof-of-concept of the importance of the soil microbial quality in the definition of “terroirs,” and more largely in the food value chain and its sustainability through microbial transfers from agroecosystems to final products. The next step will be to investigate to what extent integrating soil microbiological quality may improve the sustainability of the food production models, with projects extending this approach to other products and going further up the food value chain. To answer this question, an approach similar to the Living-lab appeared to be most promising and relevant, due to its numerous advantages for innovation in agriculture (Lacoste et al., 2022).

The “Dijon, sustainable food supply 2030” project is another example of the integration of soil quality in the food value chain at a territorial scale. This project is an ambitious and innovative ten-year plan aimed at reaching a fully sustainable agri-food model in the Dijon Métropole territory (Burgundy, France). Even though not obvious, metabarcoding of the soil DNA represents an important asset for this project. It will be implemented on a sampling of 600 points covering the entire 3,500 km^2^ of Dijon Metropole’s urban and agricultural territories, and will lead to several outputs. From a scientific point of view, the implementation of metabarcoding on a territorial scale will provide more insights into the drivers of the spatial distribution and regulation of the soil microbial diversity. From a more operational point of view, it will lead to building up the first territorial repository of the soil microbial quality in terms of microbial biomass and biodiversity. Such a repository will serve to implement a robust and sensitive diagnosis of the soil microbial quality by the actors and stakeholders involved in the use of soils (stakeholders of biomonitoring programs, farmers, land managers, agri-food industries, and consumers). This is a pre-requisite for combining food and the environmental quality of future farming systems developed in the context of the agroecological transition. Indicators and associated repositories will also be used to implement innovative labels for agricultural products, based on performance obligations rather than just best endeavors obligations. Such labels will inform the consumers about the effective environmental fingerprint of the agricultural products. They will also help move toward the development of local food systems. In addition, based on diagnosis at the territorial scale, the most sustainable farming systems and practices will be identified and communicated to the stakeholders involved in the use of soils, whether urban or rural. Soil quality indicators obtained from metabarcoding will also provide bases for soil value estimates integrating the intrinsic ecological state, which has not been taken into account to date. Ultimately, the implications of this shift of paradigm will be huge in terms of soil conservation, legal terms and economic aspects. Altogether, this project illustrates to what extent metabarcoding can be a powerful tool to help in the transition of urban and rural spaces toward more sustainable systems. As a pilot project, it is aimed at being replicated in other territories in order to supply collectivities and soil users with tools for measuring the impact of their activities on the soil quality and promoting sustainable systems.

## Ongoing and Future Developments

### Recent Advanced Technologies

New technological developments frequently emerge in microbiology. Adapted from the Lawrence Livermore Microbial Detection Array (LLMDA), the Axiom Microbiome Array (AMA) is a high-throughput platform that identifies species from environmental samples. This microarray contains 1.38 million DNA probes that can detect just over 12,000 species of viruses, archaea and bacteria, fungi, and protozoa, and probably represents the most thorough method (Thissen et al., 2019).

New platforms devoted to third-generation sequencing have emerged. They provide long reads and avoid PCR steps for some technologies (Tedersoo et al., 2021). Although these technologies are increasingly used in microbial ecology research, they are not yet available for the microbial diagnosis of soils. Nevertheless, they have proved useful to characterize microbial communities in a context of diagnosis in plant pathology (Tedersoo et al., 2019). The small and large subunits of the rRNA operon can be sequenced in full length (Tedersoo et al., 2018), improving the species-level identification of prokaryote and eukaryote organisms (Benítez-Páez et al., 2016; Singer et al., 2016) and *in fine* diagnosis. The feasibility of the method was demonstrated by the Oxford Nanopore MinION TM system and PacBio sequencing with environmental samples for metabarcoding (Schloss et al., 2016; Kerkhof, 2021) and metagenomics (Van Goethem et al., 2021) studies. Real-time DNA sequencing coupled with bioinformatic analysis also appears very promising to carry out a rapid assessment of biodiversity directly in the field and might represent a reliable bio-monitoring tool in the years to come (Pomerantz et al., 2018; Krehenwinkel et al., 2019; Maestri et al., 2019; Albrecht et al., 2020).

Recent meta-omics approaches are essential to better understand the soil microbiome, but it would be interesting to also explore the microorganisms of relevance for improving the soil quality and food safety. Although significant improvements have been made in culturing so-far unculturable microorganisms, finding an appropriate culture medium remains difficult. High-throughput culturing platforms have emerged to isolate and screen a large number of microbial cells (Mafla-Endara et al., 2021; Trivedi et al., 2021). The ability to isolate single cells using microfluidics coupled with technologies amplifying genomic DNA from single cells will revolutionize the study of unculturable species and of microheterogeneity within species (Chen et al., 2021). As recently stated, microfluidics has a great potential for experimenting on the soil microbiome so as to determine the mechanisms underpinning specific microbial metabolic interactions and to understand and predict the influence of environmental gradients on specific microbial functions with precise spatiotemporal control (Jansson and Hofmockel, 2018). The latest data indicated that droplet-based microfluidic systems compartmentalize single microorganisms in droplets, detect and sort the droplets at a rate of up to 1,000 *per* second and screen ~1 million microorganisms *per* hour (Watterson et al., 2020).

### Old Data for Future Prospects, New Perspectives

Over the last decade, multiple tools or databases have been developed to maximize the use of meta-omics results. Major advances have been made in metabarcoding research, ands have provided functional information (Djemiel et al., 2022). Functional inference and functional trait assignments can be carried out in addition to the characterization of microbial diversity. Functional inference based on phylogenetic models and genomic data predicts putative bacterial and fungal functions (Douglas et al., 2020). One of the advantages is to go beyond the metagenomics approach while saving time and money. However, the major limitation is that the robustness of the results is strongly linked to amplicon resolution (corresponding to metabarcoding) and the availability of microbial reference genomes (corresponding to inference). Further tools and databases can be used to supplement microbial diversity results with trait and character data (Nguyen et al., 2016; Põlme et al., 2020). For example, based on taxonomic classification, fungal genera/species can be associated with their lifestyles, body types or habitats. Another alternative way to evaluate the quality and functioning of soil microbial ecosystems is biotic interaction network analysis (Barberán et al., 2012; Layeghifard et al., 2017). Based on the analysis of taxonomic co-abundance patterns, the topology of each network highlights the relationship between taxa – positive in the case of co-occurrence, or negative in the case of co-exclusion (Karimi et al., 2017). Exploring the keystone taxa or guilds highly connected whatever their abundance will provide a complementary approach and detect taxa with a crucial role in the soil microbiota structure and functioning (Banerjee et al., 2018), which can be modified by soil disturbances linked to agricultural intensification (Banerjee et al., 2019; Karimi et al., 2019).

## Conclusion

**Figure 8:**
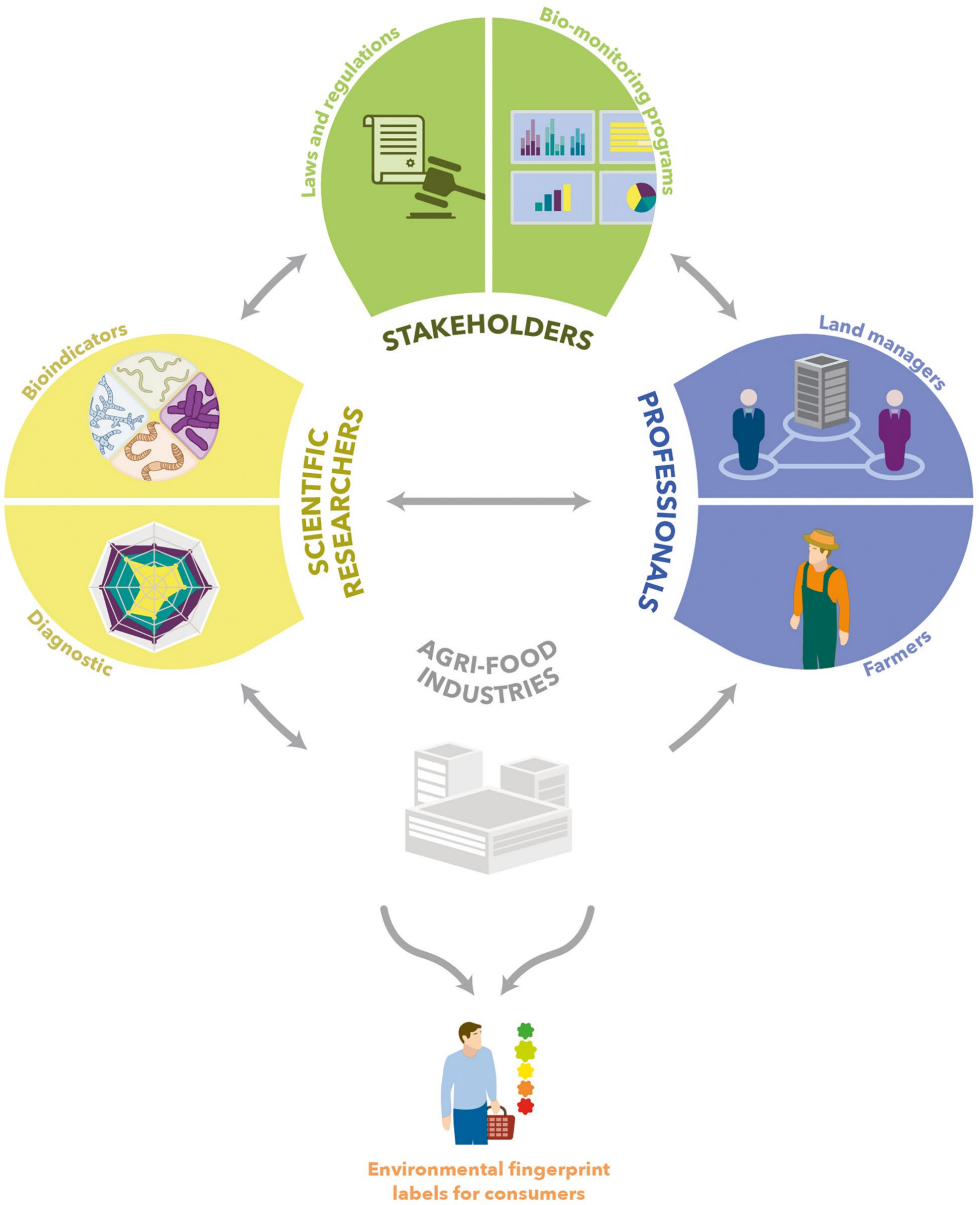
Schematic overview of field soil quality measurement and its actors. Scientific researchers explore microbial diversity in order to create repositories and develop bioindicators. These bioindicators are forwarded to the agri-food industry to make them largely accessible to professionals such as farmers or land managers. In addition, exchanges with stakeholders of bio-monitoring programs take place.

Global awareness is required for a greater consideration of microbial indicators for monitoring soil biodiversity and achieving the ambition of a sustainable food value chain. This involves a wide-scale collaboration of governments, local authorities, stakeholders of bio-monitoring programs, farmers, land managers, agri-food industries, and consumers (Lehmann et al., 2020; Fierer et al., 2021; Figure 8). Microbial indicators for soil quality monitoring by scientific researchers (Fierer et al., 2021) and routine use by field professionals (Figure 8) need to be developed and validated. Although studying microbial diversity used to be difficult owing to technical constraints, a broad range of approaches have emerged in the past few years or decades (Yang et al., 2020). Meta-omics techniques are already being used to study microbial communities along the food production chain, but insufficiently integrate the soil microbiome (Cocolin et al., 2018; Yap et al., 2022). This holistic vision is essential to offer high-value bioindicators. Moreover, robust repositories are badly needed for presently existing soil monitoring networks (Yang et al., 2020). In light of these elements, meta-omics has provided and will keep on providing modern microbial indicators for monitoring the soil biodiversity and securing food production and quality, and the related environmental footprint with the creation of labels for the consumers. This ambition to achieve sustainable food systems also applies to urban environments (Vargas-Hernández, 2020).

## Author Contributions

CD and ST conceptualized the manuscript and drafted the manuscript with contributions from SD, BK, AC, WH, ArB, AlB, SS-B, P-AM, NCP-B, and LR. All authors contributed to the article and approved the submitted version.

## Conflict of Interest

BK was employed by the company Novasol Experts.

The remaining authors declare that the research was conducted in the absence of any commercial or financial relationships that could be construed as a potential conflict of interest.

## Publisher’s Note

All claims expressed in this article are solely those of the authors and do not necessarily represent those of their affiliated organizations, or those of the publisher, the editors and the reviewers. Any product that may be evaluated in this article, or claim that may be made by its manufacturer, is not guaranteed or endorsed by the publisher.

## Supplementary Material

Supplementary Table 1 Overview of the various meta-omics approaches and their operationality for soil quality biomonitoring. R: In research phase, P: Provided by agri-food industries, XXX: Adapted technique with a very good data / price ratio, XX: Adapted technique with a moderate data / price ratio, X: Adapted technique with a low data/price ratio.
